# Intercoronary communication: a rare coronary anomaly

**DOI:** 10.1186/s43044-024-00575-2

**Published:** 2024-10-21

**Authors:** Seenu Prasanth Adimoulame, Anand Palakshachar, Rangaraj Ramalingam, Nagaraja Moorthy, Anushree Kumbhalkar

**Affiliations:** 1 All India Institute of Medical Sciences, Nagpur, India; 2 Sri Jayadeva Institute of Cardiovascular Sciences and Research, Bengaluru, India

**Keywords:** Coronary artery anomaly, Intercoronary communication, Coronary arcade, Collaterals

## Abstract

**Background:**

Intercoronary communication also known as coronary arcade or coronary cascade is a rare coronary artery anomaly with an incidence of only 0.002% in patients undergoing angiography. This case emphasizes the importance of recognizing this rare anomaly and highlights its clinical significance.

**Case presentation:**

We report a case of intercoronary communication in a 56-year-old female who presented with acute chest pain and ST-segment depression in the lateral leads. High-sensitivity troponin-T was elevated and transthoracic echocardiography revealed normal left ventricular function with no regional wall motion abnormality. Hence, the diagnosis of acute coronary syndrome – non-st-elevation myocardial infarction was considered. Coronary angiography revealed a 95% focal stenosis in the major obtuse marginal artery (OM). The right coronary artery (RCA) angiogram revealed a single abnormal channel communicating the right posterolateral branch (PLV) and the distal left circumflex artery (LCX) with retrograde opacification of the proximal LCX, left main coronary artery (LMCA) and left aortic sinus. After she underwent revascularization with the drug-eluting stent to the OM. CT-coronary angiography confirmed the presence of intercoronary communication (ICC) between the right posterolateral branch and the distal LCX artery. No active intervention was done for the ICC. Over a year of follow-up, our patient remained asymptomatic.

**Conclusions:**

Angiographically and anatomically, collaterals and intercoronary communications should be differentiated. Obstructive coronary artery disease leads to the development of collaterals, which are typically less than 1 mm in diameter, multiple and tortuous. However, ICC tends to be single and straight, usually seen without obstructive disease with unidirectional or bidirectional flow. Histologically, collaterals consist of endothelium supported by poorly organized collagen, muscle and elastic fibers. Meanwhile, ICCs resemble epicardial vessels in that they have a well-defined muscular layer. This case emphasizes the importance of recognizing this rare coronary anomaly and distinguishing it from collaterals to help in accurate diagnosis. Although they can provide an efficient blood supply to the jeopardized myocardium and can aid as a channel during coronary interventions, they can also cause myocardial ischemia by coronary steal.

**Supplementary Information:**

The online version contains supplementary material available at 10.1186/s43044-024-00575-2.

## Background

The prevalence of coronary artery anomalies in routine angiographic series is 0.3–1.3% [1–4]. One such rare anomaly is intercoronary communication, also known as coronary cascade or coronary arcade, which has an incidence of only 0.002% [1]. It is thought that a fault in the embryologic development contributed to the persistent existence of the intercoronary channel [5]. We report a case of intercoronary communication between the right coronary artery (RCA) and the left circumflex artery (LCX). This case emphasizes the importance of recognizing this rare anomaly and highlights its clinical significance.

## Case presentation

**Fig. 1:**
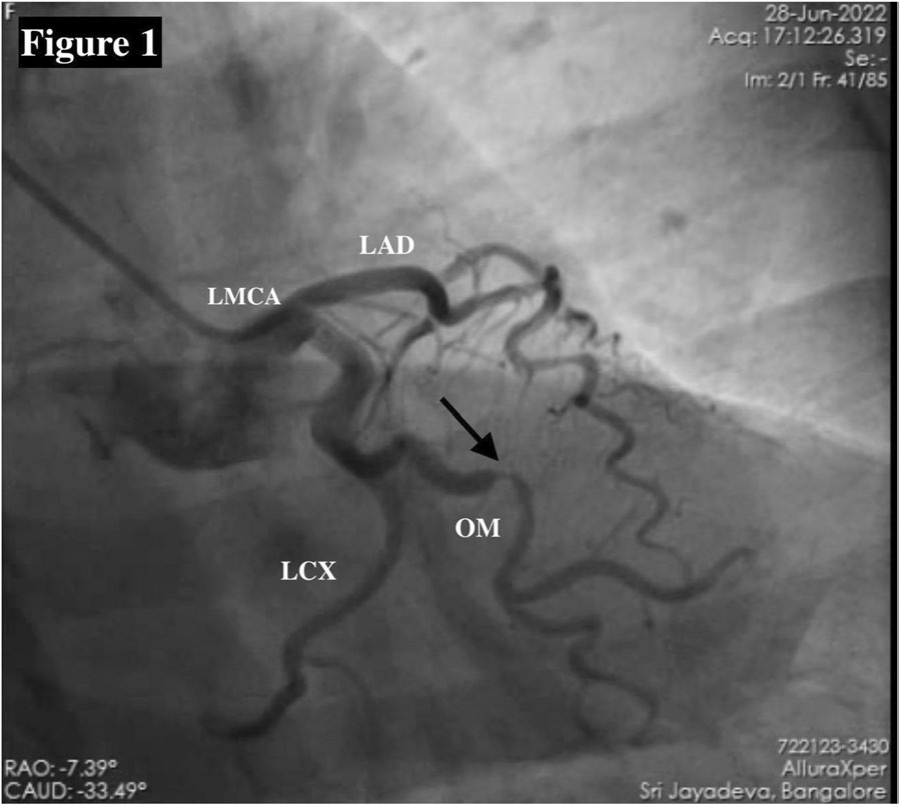
Coronary angiography of left coronary artery in RAO Caudal view showing critical discrete major OM lesion (arrow); normal left main coronary artery; tortuous but patent proximal and distal LCX

**Fig. 2:**
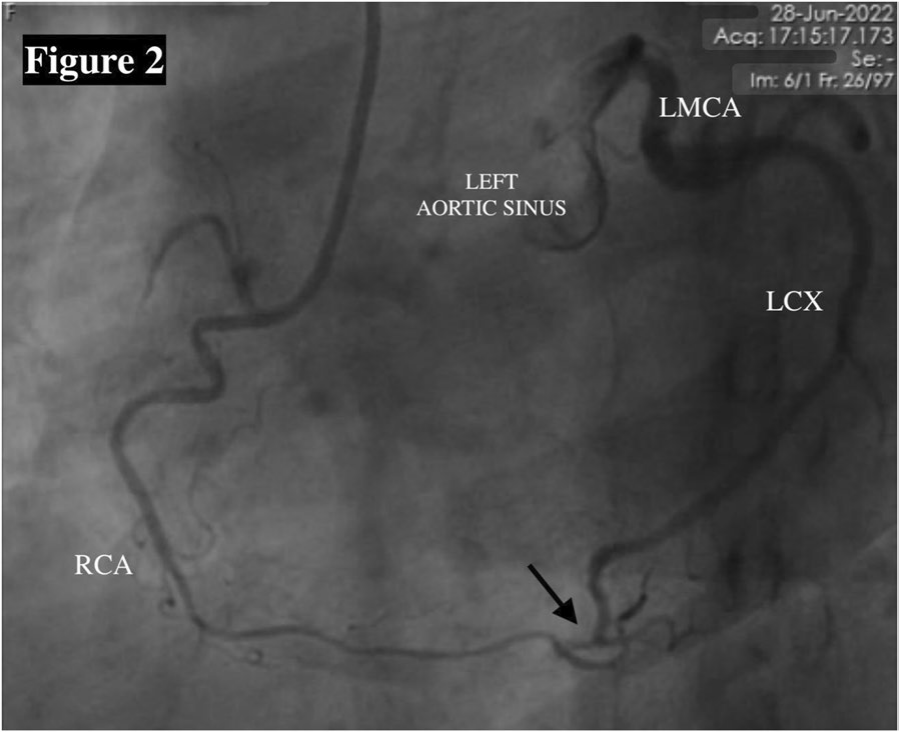
Coronary angiography of RCA in LAO view showing co-dominant RCA. There is an abnormal channel (arrow) connecting the right posterolateral branch to the distal LCX causing retrograde filling of LCX up to LMCA and left aortic sinus

**Fig. 3:**
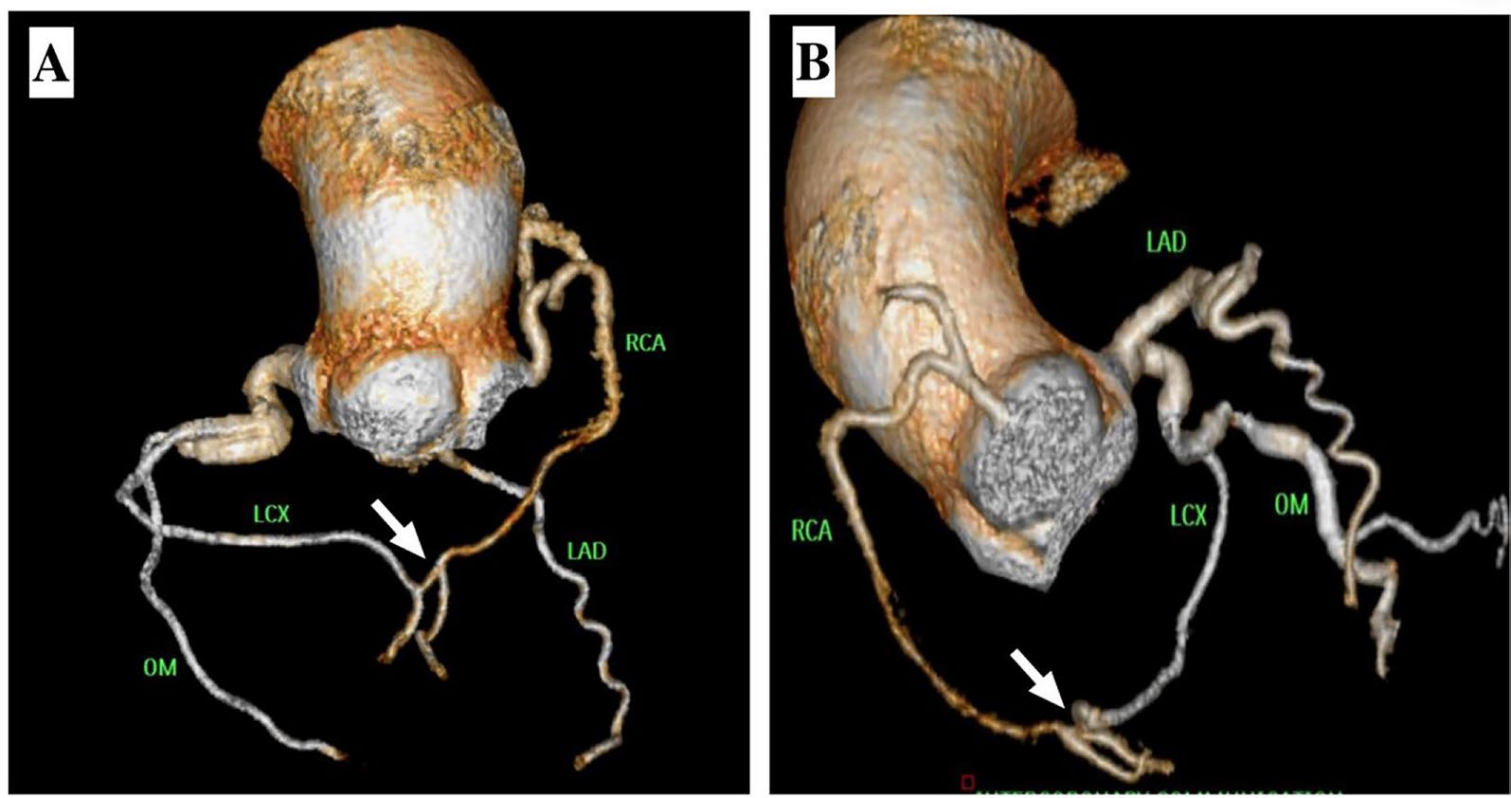
**A** and **B** Multidetector CT-coronary angiography showing an abnormal communication (white arrow) between the right posterolateral branch to the distal LCX artery

A 56-year-old woman with a history of hypertension for two years presented with acute chest pain lasting for four hours. No other significant medical, family, psycho-social history or past interventions. Her vital signs were stable, and cardiovascular and systemic examinations were normal. The electrocardiogram showed ST-segment depression on V 4-6, I & aVL. Laboratory findings showed an elevated high-sensitivity troponin-T level of 1200 ng/L (normal range: < 14 ng/L). Transthoracic echocardiography revealed mild left ventricular hypertrophy with grade I diastolic dysfunction, but no regional wall abnormality and preserved left ventricular systolic function. Hence, the diagnosis of acute coronary syndrome – non-ST-elevation myocardial infarction was considered. Coronary angiography revealed a critical discrete lesion in the major obtuse marginal artery (Fig. 1), which was stented. Otherwise, the left main coronary artery, the proximal and distal LCX was normal (Fig. 1) with mild plaque in the mid-LAD. The selective right coronary artery angiogram revealed a single abnormal channel communicating the right posterolateral branch and the distal left circumflex artery (LCX) with retrograde opacification of the proximal LCX, left main coronary artery and left aortic sinus (Fig. 2). However, the left coronary artery angiogram did not show the communication. Multidetector CT-CAG confirmed an ICC between the right posterolateral branch and the distal LCX artery (Figs. 3A & 3B). Over a year of follow-up, our patient remained asymptomatic.

## Discussion

The first reported case of the coronary arcade was in 1973 by Cheng [6]. Since then, only a few cases have been documented in the literature. In the largest study to date, Yamanaka et al. found that among 126,595 patients who underwent coronary angiography, only three individuals exhibited this anomaly, making up a mere 0.002% of the total patient population [1]. The ICCs are found in two locations: (a) in the distal part of the posterior interventricular groove, where the anterior and posterior interventricular arteries communicate, and (b) in the posterior atrioventricular groove, where the distal RCA and circumflex communicate, as in our case. [7, 8]

It is important to differentiate between collateral vessels and ICCs both anatomically and angiographically (Table 1). Obstructive coronary artery disease leads to the development of collaterals. They are typically less than one millimeter in diameter, multiple, twisted, tortuous and corkscrew shape. However, ICC is typically seen without proximal obstructive lesions and is usually single, straight or gently curved. Histologically, collaterals are made up of endothelium with poorly organized collagen, muscle, and elastic fibers. While ICCs resemble epicardial vessels in that they have a well-defined muscular layer [8]. The underlying abnormality for ICC formation has been hypothesized to be a fault in embryological development leading to the persistence of fetal coronary circulation. [5]

**Table 1:** Differentiation between collateral vessel and intercoronary communication

Collateral vessel	Intercoronary communication
Associated with proximal obstructive lesion	No evidence of proximal obstruction
Usually multiple	Usually single
Course is tortuous and twisted	Usually straight
Size is less than 1 mm	Large caliber
Histology: Composed of endothelium supported by poorly organized collagen, muscle and elastic fibers	Histology: Similar to epicardial vessel with a well-defined muscular layer

The significance of ICC depends on whether blood flow is unidirectional or bidirectional, with the ability to supply blood to the myocardium at jeopardy in the setting of stenosis [9, 10]. On the contrary, they can also cause myocardial ischemia by coronary steal [11]. Because of their larger diameter, they can deliver blood flow that is more efficient than collaterals. These can aid as a guide for navigating occluded coronaries during interventions and can also function as an optimal channel for the retrograde approach to open the occluded artery [12]. In a study of 100 human hearts which was studied postmortem, seven cases showed ICCs [13]. This shows that the anatomical prevalence of this condition is notably higher, around 7%, compared to angiographic studies with much lower prevalence of about 0.002% [1]. This suggests that these channels are usually dormant in un-diseased coronaries and become usually clinically significant when associated with obstructive CAD. [12]

Based on distinct angiographic features (single, straight, larger diameter and without proximal obstructive lesions), the abnormal communication between the RCA and LCX in our patient was determined to be congenital, and this was confirmed by multidetector CT-CAG. The unidirectional flow in our patient may have resulted from a wedged injection of the right coronary artery, which caused flow between the right coronary artery and the left circumflex artery, filling up to the left aortic sinus retrogradely, but not vice versa. Although not done, the super-selective injection of the LCX could have helped demonstrate the true one-way functionality. The assessment of hemodynamics can provide valuable insights for better understanding these distinct communications. The critical stenosis of the obtuse marginal artery which caused ischemic manifestations in our patient was addressed. Over the course of a year of follow-up, our patient remained asymptomatic.

## Conclusion

Intercoronary communication is a rare coronary artery anomaly and distinguishing it from collaterals helps in accurate diagnosis. While they can provide an efficient blood supply to the jeopardized myocardium, they can also serve as a channel during coronary interventions. However they can also cause myocardial ischemia by coronary steal. The assessment of hemodynamics can provide valuable insights for better understanding these distinct communications.

## Patient’s perspective

In 2023, March, Monday afternoon, I felt uneasiness and chest tightness. I initially felt it could be related to indigestion and took gelusil (antacids). But the pain was constant and didn’t relieve. Hence, I went to the hospital. Doctors evaluated me with ECG and blood tests. I was informed to have a heart attack and the doctors advised me to undergo an angiogram. After the angiogram, I was informed to have a block which needed a stent and also, I was also informed to have a rare abnormality in the heart vessel. After stenting, my chest pain had come down and I was able to do my routine work and got discharged in two days. Since then, I have no similar pain or discomfort. I am happy that other doctors can learn about my rare abnormality in the heart. I also give my consent to publish the related medical details.

## Supplementary Information

Additional file 1. Coronary angiography of RCA in the left anterior oblique view showing a co-dominant RCA. Note an abnormal channel connecting the right posterolateral branch to the distal LCX causing the filling of proximal LCX, LMCA and left aortic sinus retrogradely.

Additional file 2. Coronary angiography of the LCA in the right anterior oblique caudal view showing a critical (95%) discrete lesion in the major OM artery; normal left main coronary artery; tortuous but patent proximal and distal LCX. The left coronary injection does not reveal the communicating vessel.

## Data Availability

The data are available for sharing.

## Abbreviations

ICC: Intercoronary communication
LMCA: Left main coronary artery
LAD: Left anterior descending artery
LCX: Left circumflex artery
RCA: Right coronary artery
LCA: Left coronary artery
OM: Obtuse marginal artery
LAO: Left anterior oblique
RAO: Right anterior oblique
CT: Computed tomography
CT-CAG: Computed tomography coronary angiography
CAD: Coronary artery disease
